# Psoas abscess associated with aortic endograft infection caused by bacteremia of *Listeria monocytogenes*: A case report and literature review (CARE Complaint)

**DOI:** 10.1097/MD.0000000000017885

**Published:** 2019-11-11

**Authors:** Jen-Wen Ma, Sung-Yuan Hu, Tzu-Chieh Lin, Che-An Tsai

**Affiliations:** a Department of Emergency Medicine, Taichung Veterans General Hospital; b School of Medicine; c Institute of Medicine, Chung Shan Medical University; d Department of Nursing, College of Health, National Taichung University of Science and Technology; e Department of Nursing, Central Taiwan University of Science and Technology, Taichung; f Department of Nursing, Jen-Teh Junior College of Medicine, Nursing and Management, Miaoli County; g College of Public Health, China Medical University; h Department of Internal Medicine, Division of Infectious Disease, Taichung Veterans General Hospital, Taichung, Taiwan.

**Keywords:** aortic aneurysm, bacteremia, endograft infection, *Listeria monocytogenes*, psoas abscess

## Abstract

**Rationale:**

Endograft infection following endovascular stent for aortic aneurysm is rare (0.6%–3%), but it results in high mortality rate of 25% to 88%.

**Patient concerns:**

A 66-year-old hypertensive man underwent an endovascular stent graft for abdominal aortic aneurysm 18 months ago. Recurrent episodes of fever, chills, and abdominal fullness occurred 6 months ago before this admission. Laboratory data showed 20 mg/dL of C-reactive protein and abdominal computed tomography (CT) revealed an aortic endoleak at an urban hospital, so 4-day course of intravenous (IV) amoxicillin/clavulanic acid was given and he was discharged after fever subsided. He was admitted to our hospital due to fever, chills, and watery diarrhea for 1 day. Abdominal CT showed left psoas abscess associated with endograft infection. Blood culture grew *Listeria monocytogenes*.

**Diagnosis:**

Left psoas abscess associated with endograft infection caused by bacteremia of *Listeria monocytogenes*.

**Interventions:**

IV ampicillin with 8 days of synergistic gentamicin was prescribed and it created satisfactory response. Ampicillin was continued for 30 days and then shifted to IV co-trimoxazole for 12 days.

**Outcomes:**

He remained asymptomatic with a decline of CRP to 0.36 mg/dL and ESR to 39 mm/h. He was discharged on the 44th hospital day. Orally SMX/TMP was prescribed for 13.5 months.

**Lessons:**

Only few cases of aortic endograft infection caused by *Listeria monocytogenes* had been reported. In selected cases, particularly with smoldering presentations and high operative risk, endograft retention with a prolonged antimicrobial therapy seem plausible as an initial therapeutic option, complemented with percutaneous drainage or surgical debridement if necessary.

## 1 Introduction

*Listeria monocytogenes* (*L monocytogenes*) is a non–spore-forming facultatively anaerobic Gram-positive bacillus well-known for causing typical manifestations of sepsis, meningoencephalitis and endocarditis, predominantly in immunosuppressed groups such as the elderly, pregnant women, neonates, or those with defect in T-cellular immunity.^[1,2]^ Despite its predilection for bacteremia and high affinity for endovascular epithelium as well as artificial epithelium, *L monocytogenes* is rarely reported to be the causative organism of both mycotic aneurysms and vascular graft to promote awareness of the uncommon presentations of listeriosis in the English literature.^[3–14]^ First report of an aortic aneurysm caused by *Listeria* has been reported in 1965.^[15]^

We herein provide a case of psoas abscess associated with aortic endograft infection caused by bacteremia of *L monocytogenes*. This article also puts a focus on the treatment option of antimicrobial therapy alone or with additional catheter-guided drainage with preservation of the endograft, specifically for those with insidious presentations and high preoperative risk.

## 2 Case report

**Figure 1:**
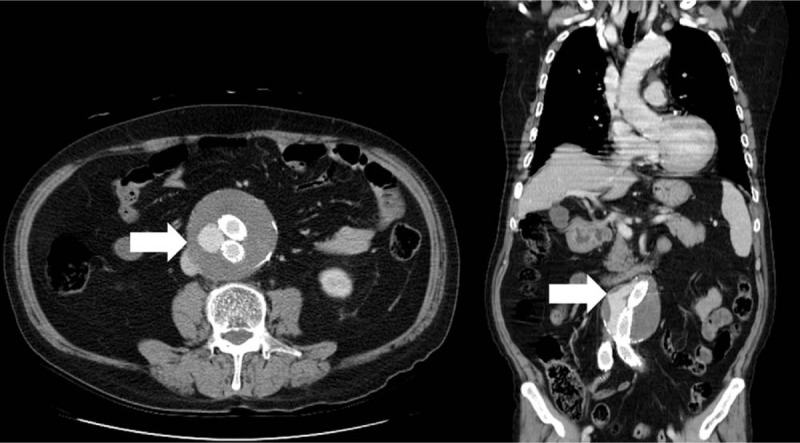
Computed tomography depicted contrast within the aneurysm sac which disclosed an endoleak (white arrow).

A 66-year-old Taiwanese man had a history of hypertension, rheumatoid arthritis on quinine, methotrexate, and methylprednisolone, and cement injection for compression fracture of osteoporotic vertebrae. He underwent an endovascular stent graft of abdominal aortic aneurysm 18 months ago at an urban hospital. According the clinical course, he had been suffering from recurrent episodes of fever, chills, and abdominal fullness 6 months ago, but no definite diagnosis could be confirmed. One month ago, his C-reactive protein (CRP) was 20 mg/dL and the contrast-enhanced abdominal computed tomography (CT) revealed an endoleak (Fig. 1), yet no fluid accumulation within the psoas muscle was detected. After a 4-day course of intravenous (IV) amoxicillin/clavulanic acid, fever subsided and he was discharged.

**Figure 2:**
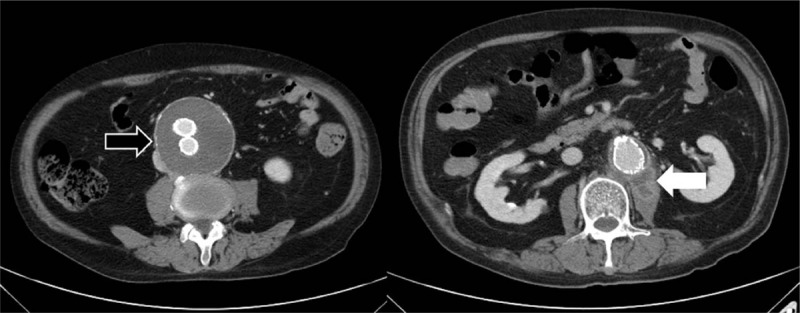
Computed tomography demonstrated a resolution of an endoleak (black arrow) and an irregular enhancement (white arrow) within left psoas muscle about 3 × 1.5 cm in size, connecting with contiguous thromboses aneurysmal sac, which was suggestive of left psoas abscess associated with an infected endograft.

This time, he presented to our emergency department with fever, chills, and watery diarrhea for 1 day. On examinations, he was febrile with a body temperature of 37.6°C, a blood pressure of 117/68 mmHg, and a heart rate of 95 beats/min. Laboratory tests revealed white blood cell count of 5700 cells/mm^3^ with segmented neutrophils of 85.5%, hemoglobin of 11.4 g/dL, platelet counts of 271 × 10^3^ cells/mm^3^, blood urea nitrogen of 17 mg/dL, keratinize of 1.1 mg/dL, sodium of 132 mEq/L, potassium of 3.7 mEq/L, albumin of 3.4 g/dL, glutamic-pyruvic transaminase of 63 U/L, alkaline phosphates of 77 U/L, lactate dehydrogenase of 381 U/L, lactate 18.5 mg/dL, CRP of 9.73 mg/dL, blood glucose of 105 mg/dL, and erythrocyte sedimentation rate (ESR) 113 mm/h. Stool analysis showed no pus cell. Abdominal CT uncovered the presence of an irregular enhancement within left psoas muscle, connecting with contiguous thromboses aneurysmal sac, which was suggestive of left psoas abscess associated with an infected endograft (Fig. 2).

After admission, cardiovascular surgeon suggested initiating cautious trial of antimicrobial therapy alone instead of concomitant radical debridement with graft removal, considering patient's frailty with immunosuppressant's and the radiological absence of endoleak and connecting fistula. CT-guided drainage was not feasible in radiologist's opinion owing to a high risk of close proximity to great vessels. The blood culture yielded growth of *Listeria monocytogenes*, susceptible to ampicillin, penicillin, and sulfamethoxazole/trimethoprim (SMX/TMP). IV ampicillin (2 g, 6 times daily) with 8 days of synergistic gentamicin (80 mg, twice daily) was chosen as the initial regiment and it created satisfactory response. Ampicillin was continued for 30 days and then shifted to IV co-trimoxazole (80/400 mg, 4 times daily) for 12 days. He remained asymptomatic with a decline of CRP to 0.36 mg/dL and ESR to 39 mm/h. He was discharged on the 44th hospital day.

Three months later, abdominal CT illustrated nearly complete resolution of bilateral psoas abscess. Orally SMX/TMP 400 mg/80 mg was prescribed for 13.5 months at outpatient follow-up. Until now, he kept regular outpatient surveillance and made an uneventful recovery.

## 3 Discussion

*Listeria monocytogenes* is a facultatively anaerobic, intracellular Gram-positive bacillus, ubiquitously been found in soil, decaying vegetation, and stool of mammals, which is mainly ingested with contaminated water and food. Approximately 1% to 5% of healthy populations are asymptomatic intestinal carriers of *L monocytogenes* and it tends to produce self-limiting febrile gastroenteritis in healthy individuals. *L monocytogenes* may migrate from human intestine into bloodstream and disseminate hematogenously to adhere to the aortic wall, then spread locally surrounding the per aortic tissues.^[1,5]^ The first choice of antibiotics is broad-spectrum penicillins like ampicillin coupled with synergistic aminoglycosides or sulfonamides.^[5]^

Endograft infection, presenting as systemic bacteremia, aortoenteric fistulae or para-aortic abscess, could result in high mortality rate (25%–88%) due to graft disruption, hemorrhage, and sepsis.^[16]^ It was once rare (0.6%–3%) but notably increasing complication for a growing number of endograft implantation as a less invasive alternative to conventional open surgery among aging population. In a retrospective multicenter analysis, total of 9739 endovascular treatments, the rate of endograft infection was 0.4% and overall mortality was 18% in 65 patients of endograft infection, particularly in conservative group (11 patients, 17%) with a mortality rate of 36%.^[17]^ Veraldi et al^[18]^ reported that the overall mortality rate was 27.9% for stent graft infection, 38.8% for conservative therapy, and 12.9% for surgical treatment. In a recent systematic review and meta-analysis consisting of 11 studies with 402 patients, 42 patients (10%) received conservative treatment, whereas 359 (90%) patients underwent surgical treatment, including removal of stent graft with in situ reconstruction or extra-anatomical bypass. Surgical group had a higher survival rate compared with conservative group (58% vs 33%).^[19]^

The presentation of endograft infection can be categorized into one-third as chronic nonspecific symptoms (malaise and weight loss), one-third as acute sepsis, and one-third as aortoenteric fistula.^[20]^ The diagnosis of graft infections, based on clinical suspicion combined with laboratory findings, imaging findings, and microbial cultures, can be challenging and often delayed due to its nonspecific symptoms. Leukocytosis and raised CRP are mostly common in laboratory findings.^[4]^ CT angiography of aorta is considered the diagnostic modality in acute stage with a sensitivity of 94% and a specificity of 85%, in which the characteristics of endograft infection are perigraft air, fluid accumulation, soft-tissue attenuation with extension into adjacent structures, pseudoaneurysm, and direct contrast extravasation in the bowel.^[21]^ Magnetic resonance imaging and a combination of positron emission tomography with ^18^F-labeled fluoro-2-deoxyglucose and CT scan possess higher sensitivity to differentiate between perigraft hematoma and subtle inflammatory changes, allowing for further confirmation of infected endografts or aneurysms.^[3,22]^ Positive blood cultures are obtained in only 21% of cases, whereas 50% cultures of drained fluid or prosthesis itself could yield growth.^[4]^ Early infection within 3 months is typically caused by *Staphylococcus aureus* (*S aureus*) and the mostly isolated microorganisms overall, from intraoperative graft contamination, are followed by *Escherichia coli*, *Enterococci*, *Salmonella*, and other GRAM-negative bacilli. Secondary infection often presents as a late complication due to haematogenous seeding from a remote source or direct extension from adjacent infected tissue.^[23,24]^ However, the microbiological epidemiology of graft infection has evolved over the years, attributable to prophylactic antistaphylococcal antibiotics, aging patients with multiple comorbidities and the increased emergent procedures, for example, methicillin-resistant *S aureus* increase in frequency and *Pseudomonas aeruginosa* account for the most Gram-negative infection now.^[2]^

The low incidence of endograft infection has not allowed establishment of a consensus on its management, warranting a case-by-case judgment dependent on the practitioner's experience. In general, the criterion standard treatment for endograft infection consists of complete graft excision, in situ reconstruction or extra-anatomical bypass, as conservative measures usually are associated with poor outcome.^[16,25]^ Conservative treatment without endograft removal, mainly appropriate antimicrobial therapy combined with radiological drainage or surgical debridement, is often reserved for patients with high perioperative risk or those presenting indolently, such as a bacteremia without shock, active hemorrhage, pseudoaneurysm, and significant endograft contamination.^[6,16,26]^ Some authors suggest prolonged administration of antibiotics until the level of CRP returns to baseline.^[27]^

Through English literature review, only 10 sporadic cases of endograft infections with *L monocytenes* had been reported worldwide with the majority located over aorta. Among the total 11 cases (Table 1), including our current report, complete resection of the affected graft was chosen as the first step in only 3 cases, whereas in the other 8 cases it was started on antimicrobial therapy along with graft preservation, 6 cases of which obtained favorable outcome, including 2 cases subsequently necessitating percutaneous drainage and surgical debridement. With 2 exceptions for 10 and 4 years, the duration between endograft implantation and clinical infection was no longer than 2 years. Overall, 10 of the 11 reported cases survived, and the only 1 who eventually died of pneumonia was in the group receiving removal of endograft infection at first.

**Table 1:**
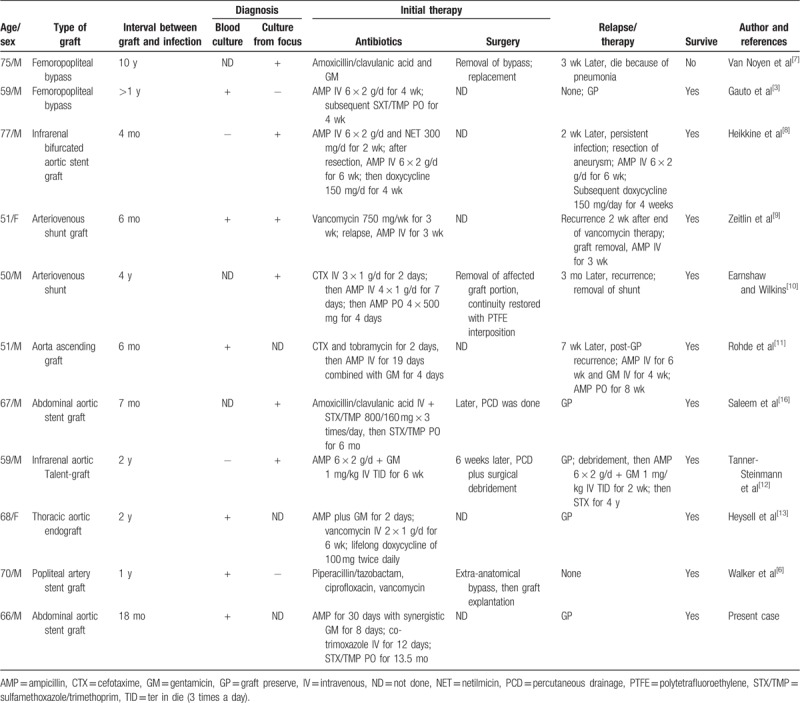
Cases and clinical characteristics of endograft infection caused by bacteremia of *Listeria monocytogenes*.

## 4 Conclusions

Although controlled trials do not exist to guide management, the criterion standard therapy remains complete graft explantation with replacement by autologous material or extra-anatomic reconstruction. As a growing mass of reports describing graft retained with desirable outcome, we propose that in selected cases with smoldering presentations and high operative risk, endograft retention with a prolonged antimicrobial therapy seem plausible as an initial therapeutic option, complemented with radiological drainage or surgical debridement tailored to the clinical condition and close monitoring the level of CRP.

## Acknowledgments

The authors express their gratitude for the tremendous efforts of the emergency resuscitation team, the radiological technicians, and the intensive care unit in the clinical diagnosis and management of this patient.

## Author contributions

**Conceptualization:** Jen-Wen Ma, Sung-Yuan Hu.

**Data curation:** Jen-Wen Ma, Sung-Yuan Hu, Tzu-Chieh Lin, Che-An Tsai.

**Supervision:** Sung-Yuan Hu, Tzu-Chieh Lin.

**Writing – original draft:** Jen-Wen Ma, Sung-Yuan Hu.

**Writing – review & editing:** Sung-Yuan Hu, Tzu-Chieh Lin.
